# Social Learning in Horses—Fact or Fiction?

**DOI:** 10.3389/fvets.2018.00212

**Published:** 2018-09-06

**Authors:** Maria V. Rørvang, Janne W. Christensen, Jan Ladewig, Andrew McLean

**Affiliations:** ^1^Department of Biosystems and Technology, Swedish University of Agricultural Sciences, Alnarp, Sweden; ^2^Department of Animal Science, Aarhus University, Tjele, Denmark; ^3^Department of Veterinary and Animal Sciences, University of Copenhagen, Copenhagen, Denmark; ^4^Equitation Science International, Tuerong, VIC, Australia

**Keywords:** cognition, equine, local enhancement, social facilitation, social learning, social transmission, training, welfare

## Abstract

Prima facie, the acquisition of novel behaviors in animals through observation of conspecifics seems straightforward. There are, however, various mechanisms through which the behavior of animals can be altered from observing others. These mechanisms range from simple hard-wired contagious processes to genuine learning by observation, which differ fundamentally in cognitive complexity. They range from social facilitation and local enhancement to true social learning. The different learning mechanisms are the subject of this review, largely because research on learning by observation can be confounded by difficulties in interpretation owing to the looming possibility of associative learning infecting experimental results. While it is often assumed that horses are capable of acquiring new behavior through intra-species observation, research on social learning in horses includes a variety of studies some of which may overestimate the possession of higher mental abilities. Assuming such abilities in their absence can have welfare implications, e.g., isolating stereotypical horses on the assumption that these behaviors can be learned though observation by neighboring horses. This review summarizes the definitions and criteria for the various types of social transmission and social learning and reviews the current documentation for each type in horses with the aim of clarifying whether horses possess the ability to learn through true social learning. As social ungulates, horses evolved in open landscapes, exposed to predators and grazing most of the day. Being in close proximity to conspecifics may theoretically offer an opportunity to learn socially, however anti-predator vigilance and locating forage may not require the neural complexity of social learning. Given the significant energetic expense of brain tissue, it is likely that social facilitation and local enhancement may have been sufficient in the adaptation of equids to their niche. As a consequence, social learning abilities may be maladaptive in horses. Collectively, the review proposes a novel differentiation between social transmission (social facilitation, local, and stimulus enhancement) and social learning (goal emulation, imitation). Horses are undoubtedly sensitive to intra-species transfer of information but this transfer does not appear to satisfy the criteria for social learning, and thus there is no solid evidence for true social learning in horses.

## Introduction

Individual behavior can be altered in various ways, some of which involve actual learning mechanisms, while others such as social facilitation rely on a particular behavior being contagiously triggered by a similar behavior in others (e.g., flight responses in horses or yawning in humans). Conversely, learning of novel behaviors can occur through individual or social learning. Individual learning refers to the animal acquiring new behavior by the trial-and-error processes of associative learning, that is learning by its own experience. In contrast, the contemporary understanding of social learning is that the animal attains new behavior after observing a conspecific performing the behavior (1). At least some forms of social learning are likely to entail higher mental abilities such as insight as they require the animal to see and remember the behavior, transfer the behavior to its own behavioral repertoire, and subsequently perform it (2). In theory, social learning is related to group-living because living in close proximity to conspecifics offers an opportunity to watch and learn (3, 4). Several authors have also emphasized this connection [e.g., (5, 6)], although more recent studies have reassessed the theory and found conflicting results, possibly due to interspecific differences in learning [e.g., (7)].

As group-living animals, it is often assumed that horses are capable of acquiring new behavior through observation of conspecifics (8–10), but solid evidence of true social learning in horses is lacking. Research on social learning in horses includes a variety of studies, some of which may over-estimate the mental abilities of horses. This review critically assesses these studies and reveals that the number of demonstrations provided by the demonstrator horse varies to the extent that one could reasonably argue that the observer horses learned by *associative* learning rather than by *social* learning. Importantly, over-estimating the mental abilities of horses is not only a scientific challenge but may also have welfare consequences. The anecdotal assumption that horses can learn so-called “vices” via observation of conspecifics is often used as an argument to keep stereotypic horses in social isolation to prevent the abnormal behavior from spreading (11, 12). This assumption, however, has never been confirmed in either experimental (13, 14) or in epidemiological studies (15). Additionally, horse trainers may assume that naïve horses are able to observe and learn from older, well-trained horses (9, 16). Although horses undoubtedly are sensitive to transfer of emotional states (17) they are less likely to learn specific behaviors from conspecifics. Both over- and underestimating the mental capabilities of horses can have significant welfare implications as this has been used to justify punishment in some training systems. Training methods based on a flawed understanding of equine learning processes may to some extent explain differences in horse training methodologies [reviewed in (18)].

Dennett (19) pointed out that assuming without solid proof that animals have insight into their instinctive behaviors amounts to an unacceptable rejection of the null hypothesis. With regard to the implication of higher mental abilities in animals where such abilities may not be within the cognitive realm of the subject species, a similar precautionary principle should be applied.

This review aims to assess the evolutionary basis for higher mental abilities in horses, including their ability to learn new behavior from observation of conspecifics. We revisit studies on social learning in horses and discuss the extent to which the available results may reflect social influence on individual learning, rather than true social learning.

## Definitions of social cognitive mechanisms

“In no case is an animal activity to be interpreted in terms of higher psychological processes if it can be fairly interpreted in terms of processes which stand lower in the scale of psychological evolution and development” (20).

Lloyd Morgan's Canon is a fundamental tenet in cognitive science. Morgan was reacting to interpretations of animal behavior he found excessively anthropomorphic and described cases in which behavior that may, at first, seem to involve higher mental processes could in fact be explained by simple associative learning. He used the example of how his dog skillfully opened the garden gate, which could easily be interpreted as an insightful act by someone seeing only the final behavior. Morgan had, however, recorded the series of approximations by which the dog gradually learned the response and could demonstrate that no insight was required to perform the behavior.

In line with Lloyd Morgan's observations, humankind's general fascination with the cognitive abilities of animals may sometimes lead to conclusions that ascribe higher mental processes to animals than are actually necessary to perform a specific behavior. For example, doubts were raised on imitation as the underlying mechanism for two of the most well-known examples of apparently imitative behavior transmission: potato washing in Japanese macaques (21) and milk bottle opening in tits (22). Reinforcement by human caretakers may have interfered with the spread of potato-washing, which was found to be easily learned by monkeys (21). Similarly, Sherry and Galefs' (22) results demonstrated that experience with previously opened bottles was sufficient to establish milk bottle opening in birds, i.e., no observation of a demonstrator bird was necessary.

Since Edward Thorndike set out to investigate whether animals can “*from an act witnessed, learn to perform that act”* (23), a considerable amount of research has aimed at exploring the cognitive abilities of animals to learn via observation of conspecifics. The term ***social learning*** has been used in a broad and general way to label a wide range of cognitive processes. Some of these only include mere social influence on individual learning, e.g., local enhancement, while others such as the more complex processes of imitation and goal emulation require higher mental abilities (24, 25). To avoid the misleading implication of higher mental abilities across the gamut of so-called social learning, we suggest a more precise taxonomy. We suggest that social influence on individual learning is clearly distinct from true observational learning and should instead be labeled ***social transmission***, i.e., a transfer of information between individuals that merely influences the likelihood of subsequent individual learning.

Henceforth, in this review we use the term ***social transmission*** to cover all processes that involve a more simple transfer of information and/or behavior between individuals of the same or different species, whereas ***social learning*** encompasses observational learning of novel behavior requiring more complex cognitive abilities. We use the term ***social learning*** to describe intra-species processes, i.e., learning from conspecifics, and the more general term ***observational learning*** to refer to inter-species processes, i.e., learning from observation of an individual of another species. In this dichotomy, ***social transmission*** includes the following terms:

- ***Social facilitation***, in which the behavior of a conspecific changes the *motivation* of the observer, resulting in the tendency of individual animals to do what other individuals are doing. This phenomenon can be considered a social influence on behavior but not as a form of learning, as it only leads to an increase (or decrease) in performance of an existing behavior (26, 27). Social facilitation is involved in the synchronization of various behaviors, such as feeding and resting behavior (28).
- ***Stimulus enhancement***, where the observer becomes more likely to interact with stimuli of the *same physical type* as those with which the demonstrator interacts. The observer is therefore more likely to learn about the *consequences* of interacting with these types of stimuli through individual associative learning (1, 27, 29).
- ***Local enhancement***, where the behavior of a demonstrator results in an increase in the *salience* of a particular stimulus or location. The observer's attention may be increasingly drawn toward previously irrelevant features, or the observer's motivation to investigate the stimulus or location may be increased. Any subsequent acquisition of the same motor behavior as the demonstrator will be accomplished by individual associative learning directed toward the newly salient part of the environment (30, 31).

In contrast, ***social learning*** requires higher mental abilities and includes:

- ***Goal emulation***, which refers to the reproduction of the *results* of a model's behavior, rather than the reproduction of the precise *behavior* that produced those results. The observer sees the movement of the objects involved and then gains new insight about their relevance to its own motivations (32).

- ***Imitation***, which describes situations where the observer *copies* the motor patterns of the demonstrator by some process of cross-modal matching (31). Imitation of non-vocal demonstrations requires an observer to match a visual representation of an observed motor input with its own proprioceptive control and regardless of the concurrent visual signal of its own behavior (25). Thus, imitation requires a certain cognitive sophistication. It is notable that many of the classic field observations of apparent imitation could be explained as examples of associative learning, mediated by local or stimulus enhancement or even explicit human reinforcement (25).
- ***Program-level imitation***, which refers to the most cognitively complex expression of observational learning. It involves a *sequence of copied movements* that are observed and imitated (33). Program-level imitation evolved for the rapid acquisition of complex skills and is seen in animals such as mountain gorillas where the young learn how to prepare certain noxious plants for consumption.

The first convincing evidence of imitation in animals came from studies reporting that naive budgerigars (observers) that had watched a trained conspecific (a demonstrator) use either its foot or its beak to press a lever to obtain food tended to use the same appendage as had their respective demonstrators (34). Later, a number of other authors conducted “two-action experiments” and reported similar effects, e.g., rats pushed joysticks either to the right or left, depending on the act of the demonstrator (35) and chimpanzees either pulled or pushed artificial fruit to obtain rewards (36). These studies provide evidence that by observing an act some animals tend to produce that same act. However, Galef (24) argues that since the behaviors needed to perform the acts (stepping, pulling, pushing) were already present in the animals' behavioral repertoires, the two-action experiments only provide evidence that observing an act can increase the relative probability that an animal will express that act rather than others in its repertoire. Instead, Galef (24) argues, true imitation requires that the animals copy a completely novel behavior, which was not previously in its behavioral repertoire.

Whereas, social learning in terms of imitation of motor patterns may play an important role in the acquisition of new skills in some species, it is noteworthy that social transmission in a broader context has a variety of functions, e.g., in relation to acquisition of information about the environment and the acquisition of social behavior (31). In this context, local or stimulus enhancement followed by associative learning may be a more efficient way of acquiring skills in many circumstances. As we shall discuss in the next section, local enhancement appears to be more biologically relevant to horses.

## Adaptiveness of social learning in horses

The study of the adaptive use of social and non-social information has the potential to increase our understanding of how animals interact with the social and physical environments in which they live (37, 38). How animals procure their food has been a significant driving force in the evolution of mental abilities in animals and one would expect different mental abilities to have evolved for various foraging niches (39). From this viewpoint, it follows that convergent evolution of mental abilities would arise in animals that occupy similar niches. Kendal et al. (40) point out that it is more advantageous and therefore adaptive for species that use complex foraging skills, such as cooperative predation and tool-use, to rely more on social information than individual learning. In particular, the dispatching of large and dangerous prey would be ameliorated by social learning. Nevertheless, herbivores are reported to socially learn to choose food items and avoid toxic foods from a very early age (41, 42). They also monitor the eating behavior of group members and minimize the risk of predation by choosing food patches closer to conspecifics (43).

Such behaviors in herbivorous animals, however, may be more parsimoniously explained by local enhancement and associative learning. It follows that when a particular food choice is reinforced, the animal will be more likely to choose similar foods. From this viewpoint, it seems unlikely that the evolution of herbivory would require cognitive capabilities greater than local enhancement. Accordingly, Marinier and Alexander (44) have shown that foals learn their mother's diet not by social learning or even social transmission, but by coprophagy. It is evident that the horse has circumvented the cognitive complexity and energetic costliness of social learning of at least some elements of foraging via a non-cognitive process. Similarly, Provenza et al. (45) demonstrated that sheep learn food aversions via a similar non-cognitive process, which can occur even under anesthesia.

An important consideration is that herbivory may not facilitate the evolution and maintenance of higher mental abilities because of its low energy yield compared to e.g., carnivory. Brains are energetically expensive (46) and it is likely that complex mental abilities, such as those required for true social learning compared to social transmission, may be an “unaffordable luxury” for an obligate herbivore. Accordingly, this would be true for any obligate herbivore regardless of phylogenetic affinity unless it was biologically adaptive such in the Gorilla whose young learn to render stinging plants edible partly by social learning (39). As studies of the behavior of ancestral equids are scarce, studies of wild and reintroduced breeds of equids provide insight to the environment in which equine cognitive abilities evolved. Wild equids have evolved in open landscapes, exposed to predators and with high fiber/low nutrient food. Living in such an environment, synchronizing activities may help individuals increase the benefits of group living, e.g., early detection of predators and subsequent flight responses as well as forage detection when the environment is patchy (47, 48). Generally, social learning is thought to be adaptive at intermediate rates of environmental variability, because in highly variable environments, social information could be outdated or have no fitness benefit in the new environment (49). In order for social learning to be adaptive, the cost-benefit ratio of socially acquiring new skills should outweigh that of individual associative learning.

Finally, homeothermy is expensive from an energetic point of view. So too are higher mental abilities (50, 51) which are generally associated with larger brains due to more neurons and neural activity. A larger brain with more neurons functions to enhance gathering, storing and integrating information (52), and facilitating acquisition of new and altered behavior patterns through cognitive processes (51). Accordingly, Martin (53) found a significant positive correlation between basal metabolic rate and brain mass in a balanced sample of 51 mammalian species. Isler and van Schaik (50) also showed that this correlation persisted while controlling for body size effects (including 347 mammalian species). In mammals, the energy costs of homeothermy are compensated through either increased energy intake or reduced allocation of energy to other biological functions such as growth, reproduction, locomotion, or digestion (50). Clearly, homeothermy should place a limitation on costly mental processes such as those required for social learning. Indeed, as pointed out by McLean (39), this restriction is largely true of grazing mammals across the taxa, from the dual standpoint of published data on the existence of higher mental abilities and the requirements of the herbivorous niche.

On the other hand, studies on species where foraging requires refined and precise skills show much clearer signs of animals being more likely to perform a behavior after observing a conspecific performing that behavior. Voelkl and Huber (54) for instance found significant differences between the two observer groups of common marmosets, who had observed a method for opening a container by either hands or the mouth. Individuals who observed the hand method, all used their hands when opening the container, whereas individuals observing the mouth method mostly used their mouth. There are several such reports from various monkey species, but also canine species appear able to learn from observation. For example, domestic dogs observing a trained dog opening a food container with the paw, also used their paw whereas naïve dogs were more likely to use their mouth (55). Social learning is in these cases advantageous because individual learning can be costly and the advantage of exploiting the expertise of others outweigh the biological cost of this ability [for more details, see (56)].

Optimal foraging in ungulates may require little more than following the movements of other conspecifics in order to detect the best forage while local enhancement may deliver the necessary transmission of learning from one horse to another obviating the need for more energetically costly mental abilities. Thus, from the viewpoint of the equine foraging ethogram and mammalian metabolic demands, it is clear that social transmission provides sufficient transfer of behavior from one horse to another without the need for complex social learning abilities.

## Social learning or social transmission in horses?

Despite the aforementioned potential maladaptiveness of social learning in horses, a few studies have suggested that horses possess the ability to learn via this process. In one study, horses had to open a box by pulling a rope with their mouth to obtain food rewards (57). Twenty-five horses watched a trained horse demonstrating the task and in a separate experiment, 14 horses were used as controls, i.e., no demonstrations and thus possibly less food cues on the rope. The authors concluded that 12 of the 25 observing horses learned to pull the rope. However, only 4 of these horses learned the task after 8 demonstrations, three by pulling with their mouth and one by pulling with a hoof. The remaining 8 horses needed between 14 and almost 80 demonstrations to learn to pull the rope. The authors further note that the majority of the 14 control horses rapidly lost interest in the task (i.e., stopped engaging in behavior directed toward the rope) and only two learned to pull the rope after about 80 trials. The results suggest that local enhancement cues from the demonstrations gave the observing horses a small advantage compared to the controls. Additionally, the 12 horses that solved the task used different techniques in order to achieve the goal of opening the feed container, with only 6 horses using the same behavior as the demonstrator. Considering the number of horses that learned to pull the rope and the low speed at which they learned the task, the results can be more accurately explained by local enhancement and associative learning rather than social learning.

Another study analyzed the extent to which horses could learn by observation to follow a person in a round pen using four different tests (58). In the first test, 12 horse pairs were included (one demonstrator and one observer horse in each pair). The demonstrator horses either followed (*n* = 4) or did not follow the person (*n* = 8) during the demonstration. Three of the observer horses observing the following behavior, expressed following behavior themselves when subsequently tested, whereas one horse did not. None of the 8 observer horses paired with a non-following demonstrator showed following behavior when tested. In the second test, eight “dominant” horses demonstrated the following behavior to eight horses, which had participated in the first experiment and had not expressed the following behavior when tested. In this second test, the 8 horses showed following behavior when tested. In the third test, a “dominant” horse observed a “subordinate” horse perform the following behavior, resulting in one horse showing the following behavior after the demonstration whereas 13 horses did not. Lastly, the fourth test paired 8 observer horses with 8 unfamiliar demonstrator horses, which resulted in no observer horses following. Based on these tests the authors conclude that “subordinate” observers copy the behavior of a familiar, “dominant” horse, and that “dominant” horses do not copy the behavior of a familiar, “subordinate” horse. Across the tests, however, only horses experienced in round pen training (*n* = 3) performed the following behavior and as none of the inexperienced horses performed the following behavior after having watched a demonstration, it is likely that no social learning took place. Additionally, although the authors mention familiarity in their conclusions, it is unclear how familiar each demonstrator and observer was to each other: The included 38 horses were kept in groups of 11, 6, 9, and 4 horses and an additional 6 in pairs and 4 solitarily. During the experiment, 14 horses were used as demonstrators and observers, 8 horses only as demonstrators and 15 horses only as observers but without mentioning familiarity or testing for a potential group effect.

Conversely, a number of studies have failed to show social learning in horses. In a study by Baer et al. (59) observer horses (*n* = 8) watched conspecifics perform a discrimination task for 5 days with 4 demonstrations per day. Observer and control (*n* = 16) horses were then tested daily for 14 days. The discrimination learning criterion was set at 7 out of 8 responses correct with at least 5 consecutively correct. Control and observer horses did not differ significantly, but from the data it can be suggested that an effect of prior observation could have been present if more horses were included. Baker and Crawford (60) investigated if horses (*n* = 9) learned the location of grain by watching another horse finding it in one of two feed buckets of similar color and shape (i.e., only location cues). No significant difference between test and controls (*n* = 18) occurred for both first and total correct choices, nor for time to reach the feed bucket with grain. The authors therefore concluded that no social learning had occurred. In another discrimination experiment, Clarke et al. (61) tested if observer horses could learn to choose between two differently colored and shaped buckets after having it demonstrated by a conspecific. Twelve of the 14 observer horses reduced their latency to approach the bucket area during a series of 10 trials implying local enhancement, but there was no significant difference between observer and control horses in the number of correct bucket choices.

Two studies investigated the ability of horses to learn an operant task of opening a feeding apparatus by observation of a trained conspecific. One study found no significant effect of prior demonstration, only that across treatments younger horses engaged in more investigatory behavior [*n* = 18; (8)]. The other study found that horses observing a demonstrator horse opening an apparatus spent more time near the apparatus, although they did not learn to open it more quickly than control horses [*n* = 66 across two experiments; (62)]. Again, these results indicate that local enhancement cues are responsible for transfer of information between horses, rather than actual social learning. Another approach to investigating social learning in horses was conducted by Rørvang et al. (63) testing if observer horses (*n* = 11) could learn a simple detour task (turning left or right) by watching a demonstrator horse making the correct turn to navigate around a fence. Observer horses did not perform better than control horses (*n* = 11) that did not see the route demonstrated. Although turning left or right is indeed within the behavioral repertoire of horses, the test horses in this study did not appear to benefit from social observation. Thus, compared to species such as rats and budgerigars where observing an act can increase the probability that the observer animal will express that act rather than others in its repertoire (24), horses do not appear to benefit from prior social observation to solve operant and detour tasks. This difference may relate to these species facing very different foraging challenges.

Other studies have explored the effects of habituated demonstrators in fear-eliciting situations. Christensen et al. (64) investigated if a calm companion influenced fear reactions of naïve horses, by pairing 18 naïve horses with either a habituated companion horse (*n* = 9) or a non-habituated companion horse (*n* = 9). When exposed to the fear-eliciting stimulus, the horses accompanied with a habituated companion reacted less (less fear-related behavior and lower heart rate), compared to horses paired with non-habituated companions. The reduced fear reactions were also present 3 days later when the horses were tested alone (without a companion), reflecting social facilitation in combination with associative learning. Using a different experimental set-up, Rørvang et al. (65) tested the effect of prior observation of a habituated demonstrator crossing a novel surface. The observer horses watched the demonstrator crossing the novel surface from a distance of ~10 m. These observer horses (*n* = 11) had lower mean and maximum heart rates when subsequently having to cross the novel surface themselves, compared to control horses (*n* = 11), suggesting that social facilitation even occurs from a distance and with a short delay (10 s) between demonstration and test.

Studies of social learning in other domesticated ungulates support the results on social learning in horses. In cattle, Ralphs et al. (66) found that social facilitation causes naïve cattle to start eating novel and even previously avoided food items. Veissier (67) investigated if heifers were able to obtain food from a box by pressing a panel after observing a familiar conspecific doing so. Heifers observing the demonstrator were more attentive to the box and the panel but acquisition of the task did not improve. Thus, also in other herbivores such as cattle, local enhancement appears to be the underlying mechanism for exchange of information between individuals. In domestic pigs, Held et al. (68) reported that observer pigs found relocated food using fewer bucket investigations than expected by random search, after watching a demonstrator finding relocated food. Non-informed pigs were additionally able to exploit the knowledge by following behind the demonstrator pigs to the food source. Nicol and Pope (69) analyzed the extent to which pigs could acquire information from their siblings. No significant effect of observation on rewarded panel pressing was found, but pigs that had observed the demonstrators, spent more time facing the operant panels and directed more non-rewarded presses toward the operant panels compared to controls. Collectively these studies indicate an effect of social facilitation and local/stimulus enhancement on food acquisition in pigs. Additionally, in relation to food preferences, weaned piglets show a preference for a flavored feed following a 30 min social interaction with an experienced demonstrator (70), which could even override neophobia toward the feed. Thus, socially transmitted cues seem important for pig feeding behavior (71) possibly owing to their omnivorous foraging behavior (39), but nevertheless pigs do not seem to utilize actual social learning in their foraging behavior.

Horses and other domesticated ungulates are indeed sensitive to transfer of information from conspecifics and the underlying mechanisms appear to be social facilitation and local/stimulus enhancement, rather than true social learning. Notwithstanding however, there seems to be significant potential in exploring the role of the dam as a salient demonstrator to her foal in fear-eliciting situations (72, 73). Studies of this sort may help elucidate how innate fear reactions can be modulated through an appropriate maternal environment. We conclude that instead of resorting to unlikely explanations of social learning in horses in complex experimental situations, more parsimonious explanations should be sought that are consistent with the horse's evolutionary biology and the tenets of Occam's razor and Morgan's Canon.

## Conclusion and perspectives

In this review we propose a differentiation between social transmission (social facilitation, local, and stimulus enhancement) and social learning (goal emulation, imitation). The latter appears to be more cognitively complex, and in order to avoid assuming such high mental abilities of horses and for the sake of clarity of terms, this differentiation is essential. Herbivory may not facilitate the evolution and maintenance of higher mental abilities and it is likely that complex mental abilities such as those required for true social learning compared to social transmission, may be an “unaffordable luxury” for an obligate herbivore regardless of ungulate phylogenetic affinities. Studies on social transmission and social learning in horses show that horses are undoubtedly sensitive to transfer of information between conspecifics, however the underlying mechanisms are most likely to be social facilitation and local enhancement, rather than true social learning. Horse trainers should therefore not expect horses to be able to learn new behavior from watching conspecifics. Instead, acknowledging that horses are adept at using social cues in terms of social facilitation and local enhancement can greatly benefit horse training, e.g., through the use of habituated companion horses for habituation of naïve horses to frightening situations.

## Author contributions

JC, JL, and AM initiated the idea for this review and all authors contributed to the initial discussions of the subtopics of the review. MR wrote the first draft and all authors contributed in writing, proofreading, and fine-tuning the review for publication.

### Conflict of interest statement

AM is employed by company Equitation Science International, Tuerong, Victoria, Australia. The remaining authors declare that the research was conducted in the absence of any commercial or financial relationships that could be construed as a potential conflict of interest.
